# Polygonumins A, a newly isolated compound from the stem of Polygonum minus Huds with potential medicinal activities

**DOI:** 10.1038/s41598-018-22485-5

**Published:** 2018-03-09

**Authors:** Rafidah Ahmad, I. Sahidin, Muhammad Taher, ChenFei Low, Normah Mohd Noor, Chanin Sillapachaiyaporn, Siriporn Chuchawankul, Tewarit Sarachana, Tewin Tencomnao, Faizah Iskandar, Nor Fadilah Rajab, Syarul Nataqain Baharum

**Affiliations:** 1 Institute of Systems Biology, Universiti Kebangsaan Malaysia, 43600 Bangi, Selangor Malaysia; 2 Laboratory of Natural Products Chemistry, Faculty of Pharmacy, Universitas Halu Oleo, 93232 Kendari, Southeast Sulawesi Indonesia; 3 Department of Pharmaceutical Technology, Kulliyah of Pharmacy, International Islamic University of Malaysia, Jalan Istana, 25200 Kuantan, Pahang Malaysia; 4 Department of Clinical Chemistry, Faculty of Allied Health Sciences, Chulalongkorn University, Bangkok, 10330 Thailand; 5 Department of Transfusion Medicine and Clinical Microbiology, Faculty of Allied Health Sciences, Chulalongkorn University, Bangkok, 10330 Thailand; 6 Biocompatibility Laboratory, Centre for Research and Instrumentation Management (CRIM), Universiti Kebangsaan Malaysia, 43600 Bangi, Selangor Malaysia

## Abstract

Polygonumins A, a new compound, was isolated from the stem of *Polygonum minus*. Based on NMR results, the compound’s structure is identical to that of vanicoside A, comprising four phenylpropanoid ester units and a sucrose unit. The structure differences were located at C-3″″′. The cytotoxic activity of polygonumins A was evaluated on several cancer cell lines by a cell viability assay using tetrazolium dye 3-(4,5-dimethylthiazol-2-yl)-2,5-diphenyltetrazolium bromide (MTT). The compound showed the highest antiproliferative (p < 0.05) activities against K562 (Human Leukaemia Cell Line), MCF7 (Human breast adenocarcinoma cell line), and HCT116 (Colorectal cancer cells) cells. Cytotoxic studies against V79–4 cells were carried out and showed that polygonumins A was toxic at 50 µg/ml, suggesting that this compound may be used as an anticancer drug without affecting normal cells. Polygonumins A also showed promising activity as an HIV-1 protease inhibitor with 56% relative inhibition. Molecular docking results indicated that the compound possesses high binding affinity towards the HIV protease over the low binding free energy range of -10.5 to -11.3 kcal/mol. *P*. *minus* is used in Malaysian traditional medicine for the treatment of tumour cells. This is the first report on the use of *P*. *minus* as an HIV-1 protease inhibitor.

## Introduction

The use of traditional herbal medicine is widespread, and plants are sources of many natural antioxidants that might serve as leads for the development of novel drugs. Natural antioxidants and bioactive compounds derived from traditional herbal medicines have received increasing attention for their potential use in treating certain human diseases. For example, traditional herbal medicine has been used widely in cancer patients^1^ and to treat neurodegenerative disorders^2^.

*Polygonum minus*, also known as *kesum*, is an aromatic plant in the Polygonaceae family, which primarily grows in temperate regions. The plant originates from Southeast Asian countries such as Malaysia, Thailand, Vietnam, and Indonesia, where it is widely used as a spice and flavouring agent in the food industry. *P*. *minus* is abundant in Malaysia, where it has recently been listed in the National Agro-Food Policy by the Malaysian government to promote its production as a way to boost the agricultural economy. *P*. *minus* has also been recognized by the Malaysian government in the Herbal Product Blueprint as an essential oil-producing crop^3^. *Kesum* oil is a source of natural aliphatic aldehydes and could be commercially produced in North East Victoria and Australia^4^. In Japan, China, and Europe, *kesum* has long been used as a hot spice. The sprouts of *kesum* are a traditional vegetable in Japan and are often used in ‘sashimi’^5^.

In addition to its use as a spice in the food industry, *P*. *minus* has been reported for its use as a herbal medicine. *Kesum* leaf decoctions are traditionally used in indigestion relief, as ingredients in shampoo to remove dandruff^6^, and as postnatal tonics^7^. *P*. *minus* has been reported to exhibit anti-microbial activity^8^, anti-inflammatory activity^9^ and cytotoxic activity against HeLa (human cervical carcinoma) cells^10^ and gastric cytoprotective activity^11^. *P*. *minus* has also been reported to be a source of natural antioxidants for its high total phenolic content and reducing capacity^12–14^. The pharmacological properties of *P*. *minus* are based on its chemical constituents. Therefore, numerous studies on the profiling of metabolites have been performed on *P*. *minus* in the search for its active compounds^15–17^.

Encouraged by the aforementioned findings, we aimed to isolate novel bioactive compounds from *kesum* in our continued investigations on *kesum* as a medicinal plant. In the present study, we isolated a new polyoxygenated aromatic compound (polygonumins A**)** from the stem of *kesum*. Polygonumins A demonstrated cytotoxic activities against several cancer cell lines, which indicated that it has potential value in the treatment of cancers such as leukaemia. These data provide baseline information for possible use in controlling cancer diseases, particularly leukaemia. In addition, the present study was undertaken to evaluate the antioxidant and anticholinesterase activities of *P*. *minus*. We also examined the potential of polygonumins A to serve as an HIV-1 protease inhibitor. Finally, a molecular docking study was performed to support our findings, which could lead to a new drug discovery in the near future.

## Results and Discussion

### Polygonumins A structure

Spectroscopy data indicated that polygonumins A has a molecular formula of C_52_H_50_O_21_, as supported by HRESIMS [M + Na]^+^ at *m/z* 1033.2697 (1033.9642 ([M + Na]^+^ calc.) or [M]^+^ at *m/z* 1010.2697 (1010.9642 ([M]^+^ calc.).

FTIR spectra showed absorption bands at (cm^−1^) 3334.8 (hydroxyl); 2953.3 (C-H aliphatic); 1704 (C-carbonyl) and 1629.4; 1605.3, and 1513.7 (aromatics rings). ^1^H NMR spectra (acetone*d*_6_, 600 MHz) δ_H_ (ppm) 4.21 (m, 1 H, H-1a); 4.41 (m, 1 H, H-1b); 5.62 (d, 7.8, 1 H, H-3); 4.83 (ddd, 13.2, 10.2, 3.6, 1 H, H-4); 4.31 (m, 1 H, H-5); 4.61 (dd, 12.0, 3.0, 1 H, H-6a); 4.47 (m, 1 H, H-6b); 5.77 (d, 3.6, 1 H, H-1′); 4.67 (t, 7.8, 1 H, H-2′); 4.47 (m, 1 H, H-3′); 4.98 (t, 10.2, 1 H, H-4′); 4.11 (br t, 9.6, 1 H, H-5′); 4.41 (m, 1 H, H-6′a); 4.21 (m, 1 H, H-6′b); 7.55 (d, 8.4, 1 H, H-2″); 6.94 (d, 8.4, 1 H, H-3″); 6.94 (d, 8.4, 1 H, H-5″); 7.55 (d, 8.4, 1 H, H-6″); 7.57 (d, 8.4, 2 H, H-2″′/6′″); 7.57 (d, 8.4, 2 H, H-2”″/6″″); 7.55 (d, 8.4, 2 H, H-2″″′/6″″′); 6.90 (m, 2 H, H-3″′/5″′); 6.90 (m, 2 H, H-3″″/5″″), 6.94 (d, 8.4, 2 H, H3″″′/5″″′); 7.78 (d, 15.6, 1 H, H-7″); 7.72 (d, 15.6, 1 H, H-7″′); 7.68 (d, 10.2, 1 H, H-7″″); 7.64 (dd, 13.2, 1.2, 1 H, H-7″″′); 6.51 (d, 14.4, 1 H, H-8″); 6.48 (d, 13.8, 1 H; H-8″′); 6.43 (d, 12.6, 1 H, H-8″″); 6.42 (d, 16.2, 1 H, H-8″″′). ^13^C NMR spectra (acetone*d*_6_, 125 MHz) δ_C_ (ppm) 65.0 (C-1); 102.7 (C-2); 78.2 (C-3); 72.7 (C-4); 80.4 (C-5); 64.2 (C-6); 89.1 (C-1′); 73.2 (C-2′); 68.6 (C-3′); 71.4 (C-4′); 68.8 (C-5′); 63.1 (C-6′); 126.1(C-1″); 130.21 (C-2″); 115.9 (C-3″); 159.81 (C-4″); 115.9 (C-5″); 130.21 (C-6″); 130.24(C-2″′/6″′); 130.3(C-2″″/6″″); 130.4(C-2″″′/6″″′); 115.8 (3″′/5″′); 115.9 (3″″/5″″); 116.0 (3″″′/5″″′); 159.84 (C-4“′); 159.9 (C-4″″); 160.2 (C-4″″′); 169.6 (C=O^1^); 170.1 (C=O^2^); 20.0 (C-methyl^1^) and 20.1 (C-methyl^2^).

**Figure 1:**
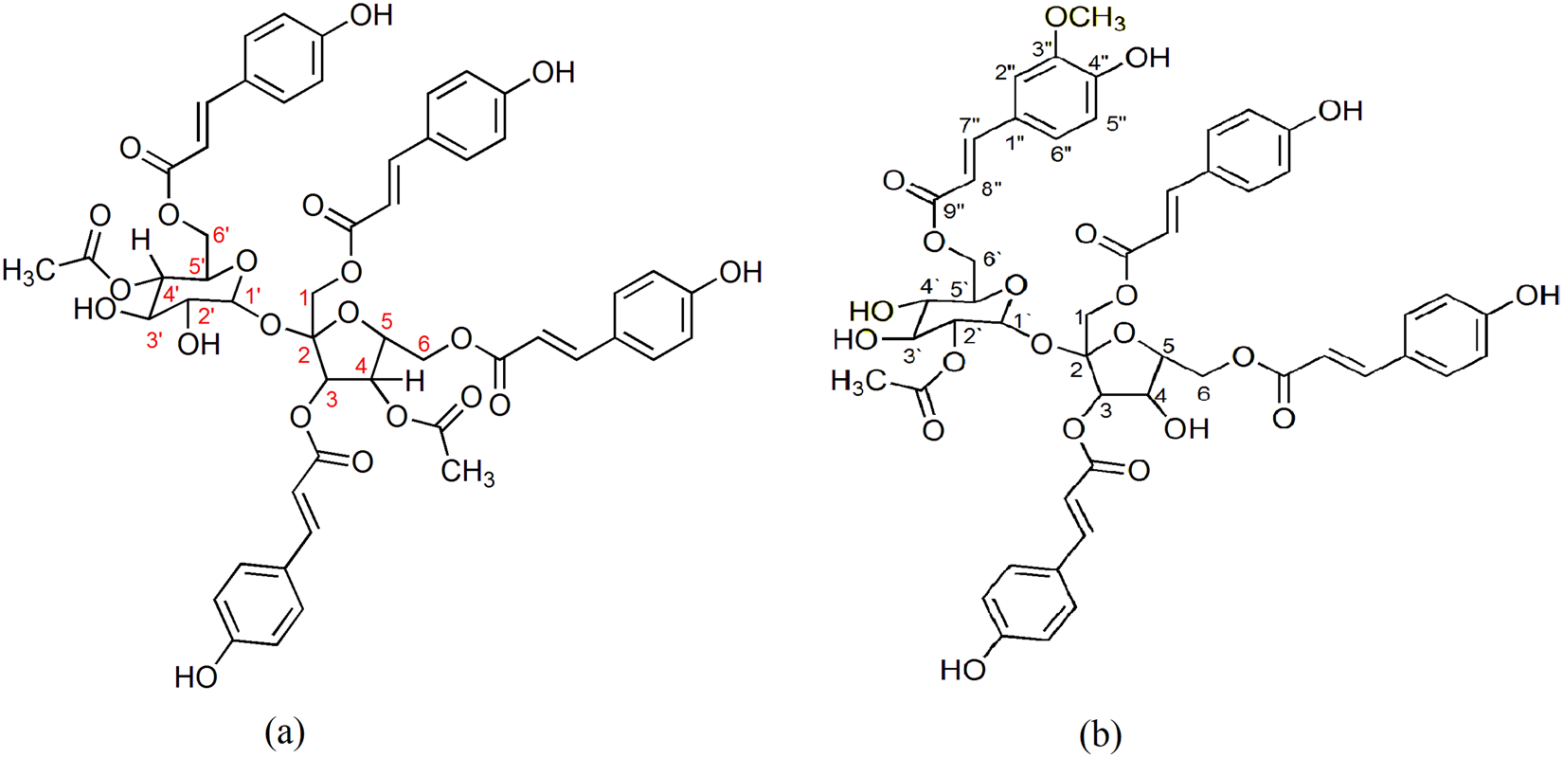
Structure of (**a**) polygonumins A (**b**) vanicoside A.

According to HRESIMS data, polygonumins A has a molecular weight of *m/z* 1010.2697 and a molecular formula of C_52_H_50_O_21_. FTIR spectra indicated that the compound has various functional groups i.e., hydroxyl absorbance at 3334.8 cm^−1^, aliphatic carbon absorbance at 2953.3 cm^−1^, carbonyl absorbance at 1704 cm^−1^ and aromatic absorbance at 1629.4, 1605.3, and 1513.7 cm^−1^. The results are supported by NMR data (^1^H and ^13^C NMR), which showed that the structure of polygonumins A comprises four phenylpropanoid units and a sucrose unit, which could be well distinguished from the structure of vanicoside A. NMR data for vanicoside A and polygonumins A are compared in Table 1 and Fig. 1.

**Table 1:** Comparison of NMR data (^1^H and ^13^C) between vanicoside A and polygonumins A.

C	Vanicoside A*		Polygonumins A	
	^1^H (300 MHz)	^13^C (75 MHz)	^1^H (600 MHz)	^13^C (125 MHz)
1	4.25, 4.6 m	63.80	4.21 (m, 1 H), 4.41 (m, 1 H)	65.0
2	—	102.12	—	102.7
3	5.61 d (8.4)	77.64	5.62 (d, 7.8, 1 H)	78.2
4	4.65 m	72.65	4.83 (ddd, 13.2, 10.2, 3.6, 1 H)	72.7
5	4.3 m	79.82	4.31 (m, 1 H)	80.4
6	4.6 m	64.55	4.61 (dd, 12.0, 3.0, 1 H), 4.47 (m, 1 H)	64.2
1′	5.66 d (3.6)	89.18	5.77 (d, 3.6, 1 H)	89.1
2′	4.69 m	73.01	4.67 (t, 7.8, 1 H)	73.2
3′	3.9 m	70.79	4.47 (m, 1 H)	68.6
4′	3.51 dd (9.4, 9.4)	70.66	4.98 (t, 10.2, 1 H)	71.4
5′	4.3 m	70.79	4.11 (br t, 9.6, 1 H)	68.8
6′	4.3, 4.35 m	64.49	4.41 (m, 1 H), 4.21 (m, 1 H)	63.1
1″, 1″′	—	125.69	—	126.1
	—	125.77	—	126.04
	—	125.84	—	125.91
	—	126.38	—	125.9
2″	7.33 d (1.8)	110.08	7.55 (d, 8.4, 1 H)	130.21
3″	—	148.97^b^	6.94 (d, 8.4, 1 H)	115.9
4″	—	147.62^b^	—	159.81
5″	6.81	114.94	6.94 (d, 8.4, 1 H)	115.9
6″	7.11 dd (1.8, 8.2)	123.24	7.55 (d, 8.4, 1 H)	130.21
7″, 7′″″	7.58, 7.62, 7.64, 7.72 ea d (16)		7.78 (d, 15.6, 1 H)	146.1
		144.92	7.72 (d, 15.6, 1 H)	145.3
		145.91	7.68 (d, 10.2, 1 H)	145.2
		145.10	7.64 (dd, 13.2, 1.2, 1 H)	145.1
8″, 8′″″	6.33, 6.40, 6.45, 6.53 ea d (16)	113.41	6.51 (d, 14.4, 1 H)	113.6
		113.87	6.48 (d, 13.8, 1 H)	114.1
		113.97	6.43 (d, 12.6, 1 H)	114.2
		114.53	6.42 (d, 16.2, 1 H)	114.3
9″, 9′″″	—	165.93	—	166.0
		165.96	—	166.04
		166.46	—	166.5
		166.64	—	166.6
2/6′″−2/6″″′	7.45–7.65 m	130.02	7.57 (d, 8.4, 2 H)	130.24
		130.06	7.57 (d, 8.4, 2 H)	130.3
		130.31	7.55 (d, 8.4, 2 H)	130.4
3/5″′−3/5″″′	6.85–6.89 m	115.63	6.90 (m, 2 H)	115.8
		115.64	6.90 (m, 2 H)	115.9
		115.73	6.94 (d, 8.4, 2 H)	116.0
4′″ − 4″″′	—	159.53	—	159.84
		159.63	—	159.9
		159.81	—	160.2
1″″″ (C=O)	—	170.12 (1)	—	170.1 (1)
2″″″	2.06 s	20.03 (1)	1.99 (s, 3 H)	20.0 (1)
1″″″′	3.85 s	55.25	—	169.6 (2)
2″″″′			1.99 (s, 3 H)	20.1 (1)
	7.33 d (1.8, OH phenolic)		7.83, 7.64 (4 OH phenolic)	
			4.21 4.41 2 (OH glucose)

**Figure 2:**
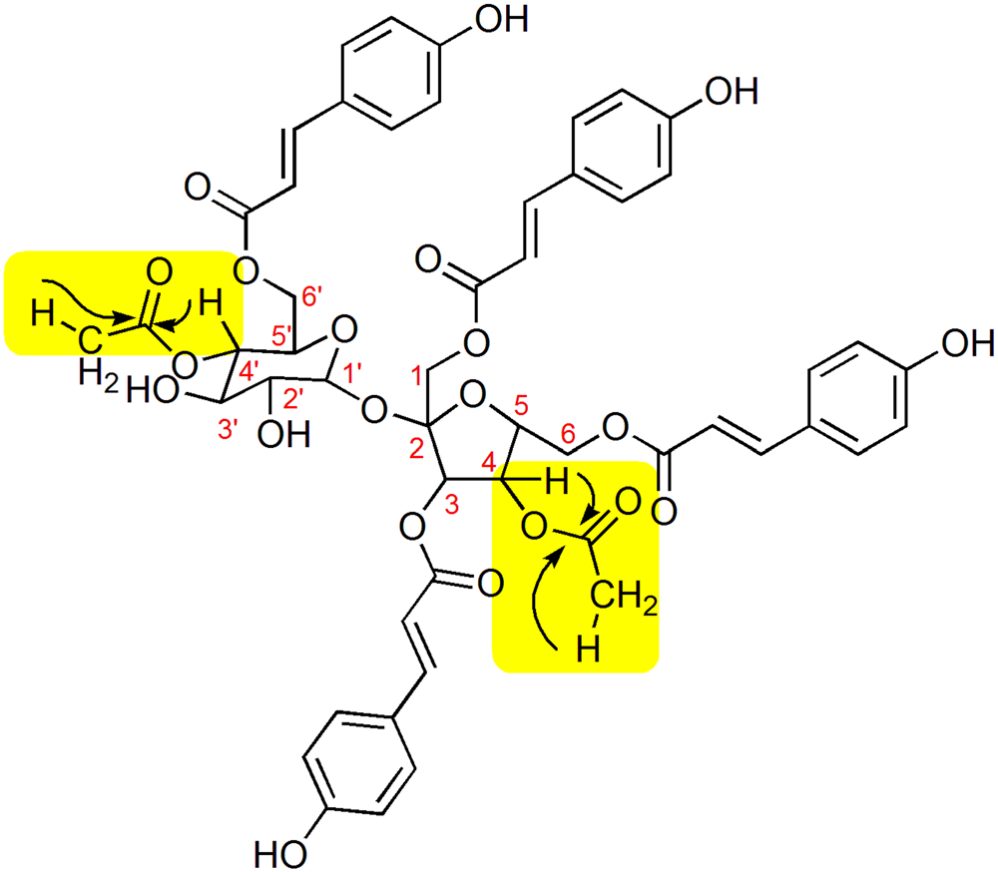
HMBC Polygonumins A.

Vanicoside A has a molecular formula of C_51_H_50_O_21_ and a molecular weight of 998 mass units^18^. The structural differences between vanicoside A and polygonumins A are located at C-3″″′. Carbon C-3″″′ of vanicoside A binds a methoxyl unit with δ_C_ and δ_H_ chemical shifts of 55.25 ppm and 3.85 ppm, respectively. Carbon C-3″″′ of polygonumins A binds a proton unit with a chemical shift δ_H_ of 6.94 (*d*, 8.4, 1 H). Furthermore, structural differences between vanicoside A and polygonumins A are observed for the substituents at C-4, C-2′ and C-4′. In vanicoside A, the substituents at C-4, C-2′ and C-4′ are hydroxyl, ethanoyl and hydroxyl units, respectively. However, the substituents at C-4, C-2′ and C-4′ in polygonumins A are ethanoyl, hydroxyl, and ethanoyl units, respectively. In the HMBC spectrum of polygonumins A in particular, the first ethanoyl unit shows long-range ^1^H-^13^C correlations between the signal δ_H_ 4.98 ppm (H-4′) and the carbonyl signal δ_C_ 170.1 ppm (C-2′″″″) and between the signal δ_H_ 1.99 ppm (H-1″″″′) and the carbonyl signal δ_C_ 170.1 ppm (C-2′″″″). The second ethanoyl unit of polygonumins A shows long-range ^1^H-^13^C correlations between the signal δ_H_ 4.83 ppm (H-4) and the carbonyl signal δ_C_ 169.6 ppm (C-2″″″) and between the signal δ_H_ 1.99 ppm (H-1″″″′) and the carbonyl signal δ_C_ 169.6 ppm (C-2″″″) (Fig. 2).

### Biological activities

It has been recommended that in selecting plant medicines for cancer treatment, ethnopharmacological uses in cancer-relevant diseases such as inflammation, infection, and immune and skin disorders should be taken into account^19^. In the present investigation, polygonumins A, a novel compound isolated from the stem of *P*. *minus*, was first evaluated for its antiproliferative activities against HCT116, C33A, H1299, MCF7, A549, and K-562 cancer cell lines using the MTT assay and then for its activities in radical scavenging and acetylcholinesterase inhibition. The activity towards HIV-1 protease inhibitor was also evaluated, as the newly isolated compound has a structure similar to that of vanicoside A.

The IC_50_ values inhibiting cell proliferation were determined, and the results are summarized in Table 2. The results showed that polygonumins A exhibited cytotoxicity against all tested cell lines except for the A549 cell line. Considering the cutoff point of 4 µg/ml for potential anticancer compounds^20^, values below this set point were obtained for this compound in HCT116 (IC_50_ 3.24 µg/ml), K562 (IC_50_ 2.25 µg/ml) and MCF7 (IC_50_ 2.87 µg/ml), indicating that polygonumins A could be defined as an anticancer compound. Polygonumins A was most effective against K562 (human leukaemia cell line), with an IC_50_ of 2.25 µg/ml, lower than that of doxorubicin (2.97 µg/ml), highlighting the anti-leukaemia potential of polygonumins A. It has been well observed that leukaemia is more sensitive to chemotherapy than other malignancies are^21,22^.

**Table 2:** Biological activities of polygonumins A, vanicoside A and vanicoside B.

	PolygonuminsA		Vanicoside A	Vanicoside B
Biological activity		IC_50_ (µg/ml)^a^		
Cytotoxic activity	HCT116 Doxorubicine^b^ C33A Doxorubicine H1299 Doxorubicine MCF7 Doxorubicine A549 Doxorubicine K562 Doxorubicine V79-4 cells	3.24 ± 0.73 2.97 ± 0.17 36.84 ± 7.16* 0.90 ± 0.1 4.71 ± 0.68* 10.12 ± 0.35 2.87 ± 0.28* 0.52 ± 0.1 — 3.13±0.11 2.25 ± 0.24 2.13 ± 0.54 >50	—	Inhibitory effects on two-stage carcinogenesis of mouse skin tumor^48^
			Compound isolated from *Polygonum pensylvanicum* showed Cyototoxic activity againts MCF cell line^18^	Compound isolated from *Polygonum pensylvanicum* showed Cyototoxic activity againts MCF cell line^18^
Antioxidant Activity (DPPH scavenging activity) (Total Phenolic Content (mg GAE/g) (Reducing Power Assay (EC50 (µg/ml))	Polygonumins A Gallic acid^b^ Ascorbic acid^b^ Polygonumins A Polygonumins A Gallic acid	812.83 ± 41.11* 35.48 ± 1.03 63.12 ± 1.11 124.0625 ±0.88 89.3 ± 2.3 59.3 ± 0.58	Isolation from *Polygonum hydropiper* showed significant DPPH scavenging activity at 26 μg/ml^49^ —	—
Anticholinesterase activity	Polygonumins A Tacrine^b^	1980 ± 25.02* 2.54 ± 0.01	—	Anticholinesterase activity at 90 μg/ml was reported in *Polygonum sachalinensis*^33^
α-Glucosidase Inhibitory Activity	n.d			Inhibitory activity was observed at 225 μg/ml in *Polygonum sachalinense*^33^
β-glucosidase Inhibitory activity	n.d		IC 50 of 59.8 μg/ml was observed in *Polygonum sachalinense*^50^	IC 50 of 48.3±1.39 was observed in *Polygonum sachalinense*^50^
Antimicrobial activity	n.d		Compound isolated from *Polygonum sachalinense* showed antimicrobial activity againts fish pathogen *Photobacterium damselae* subsp. *piscicida*^51^	Isolated from *Polygonum sachalinense* showed antimicrobial activity againts fish pathogen *Photobacterium damselae* subsp. *piscicida*^51^

^a^Data are expressed as mean ± SEM of three independent experiments; ^b^Positive control.

n.d not determined.

*Significant p < 0.05 between positive control and tested compound.

In addition to its sensitivity towards the leukaemia cell line, polygonumins A exhibited cytotoxic activities against human breast cancer and colorectal cancer cells. This finding could well explain why polygonumins A has been widely used to treat digestive disorders. It is believed that the sugar moiety, a sucrose unit, in its structure plays an important role in determining its pharmacological and biological activities. Structure and activity relationship (SAR) analysis of several related compounds containing sugar moieties, such as CCL-34 and its natural analogues, revealed that the sugar moiety was essential to their anticancer activity^23^. In particular, the sugar moiety was recognized to be critical to the topoisomerase inhibition activity of anthracyclines as antitumour drugs. It has also been suggested that the sugar structure in daunorubicin plays a critical role in determining its anticancer activity^24^. To date, there is no report on the cytotoxicity activity of vanicoside A, except in an MCF cell line. However, vanicoside A has been classified as a member of the family of protein kinase C inhibitors^18^, which exhibit antitumour effects.

The equivalence between the therapeutic effect on cancer cell lines and the toxicological effect on target human organs is an important criterion of the applicability of an anticancer compound. Therefore, we conducted toxicological studies on the V79–4 cell line (lung fibroblast cell line derived from Chinese hamsters) to determine the safe concentration range of polygonumins A. This test has been widely used to evaluate general cytotoxicity and target organ toxicity^25^. In some cases, this test may also provide information about lethal dose *in vivo*^26^. The results indicated a loss of viability of V79–4 fibroblasts at concentrations above 50 µg/ml following approximately 24 hours of exposure. Thus, the compound is approximately 10 times as toxic towards cancer cells and normal cells at these concentrations, as it is at its therapeutic concentration.

Interestingly, vanicoside A also showed significant β-glucosidase inhibitory activity, indicating promising therapeutic potential in the treatment of metastatic cancer and human immunodeficiency virus infection (HIV)^27^. As polygonumins A possesses a structure similar to that of vanicoside A, we performed an HIV-1 protease inhibition test against the newly isolated compound. Based on our results (Table 3), polygonumins A showed potential activity as an HIV-1 protease inhibitor. A relative inhibition level of 56% towards HIV-1 protease was detected compared with pepstatin A as a positive control. This is the first report indicating that *P*. *minus* exhibits activity towards HIV-1 protease. Several *Polygonum* spp have been reported to possess anti-HIV properties, with compounds such as flavonoid glycoside, quercetin and phenolics playing an essential role in anti-HIV activity^28–30^. Based on the results of this study, we believe that the phenyl propanoid glycoside moiety in the structure of polygonumins A is associated with the activation of anti-HIV protease activity. Moreover, previous study have shown that the phenylpropanoid glycoside group acts as an inhibitor of HIV-1 integrase activity, thus supporting our findings^31^

**Table 3:** Relative inhibition of polygonumins A against HIV-1 protease.

	% relative inhibition^a^
Pepstatin A (1 mM)	81.48 ± 0.76
1% DMSO	8.07 ± 0.13
Polygonumins A	56.51 ± 0.13

^a^Data are expressed as mean ± SEM of three independent experiments.

Several techniques have been used to determine antioxidant capacity. One of the methods we used is based on scavenging activity, in which we measured the ability of polygonumins A to donate electrons to a free radical to scavenge potential damage. The antioxidant activity determined by the DPPH free radical-scavenging method (Table 2) was not consistent with our expectations. The measured IC_50_ value of 812 µg/ml indicated that polygonumins A is far less potent than gallic acid and ascorbic acid. It has been reported that ethanolic, methanolic, and aqueous extracts of *P*. *minus* exhibit antioxidant activity as remarkable as that of gallic acid and ascorbic acid^13,32^. This activity is due to the phenylpropane group of *P*. *minus*. The four units of the phenylpropanoid group in the structure have been show to produce lower scavenging activity^33^. However, the high antioxidant activity of *P*. *minus* extracts has been attributed to high contents of polyphenolic compounds^16^. Therefore, we measured the total phenolic content of polygonumins A by the Folin-Ciocalteu method. Phenols consist of hydroxyl groups that are able to destroy free radicals to form stable phenoxyl radicals^34^. Although the results of the DPPH assay showed a weak scavenging ability, the total phenolic content of this compound is relatively high at 124.0625 ± 0.88 mg GAE/g when compared with that of crude extract. In our previous studies, we found that the total phenolic content in crude *P*. *minus* ethanolic extract produced the highest scavenging activity, ranging from 100 to 140 mg GAE/g^16^. Perhaps the antioxidant activity of this compound is not due to DPPH scavenging activity alone. Because antioxidant activity can be realized by multiple mechanisms or a single mechanism, we analysed the reducing power capacity of polygonumins A. This assay determines a substance’s ability to reduce Fe^3+^ to Fe^2+^. The presence of antioxidants in the extracts result in the reduction of the ferric cyanide complex (Fe^3+^) to the ferrous cyanide form (Fe^2+^). We found that polygonumins A may act as an electron donor (a hydrogen electron donor), supporting its antioxidant activity, as the compound showed a comparatively high reducing capacity, with an EC_50_ of 89.3 µg/ml, near the value of its reference compound gallic acid (Table 2). The reducing power of this compound increased rapidly with its concentration. Based on this antioxidant activity, the compound’s high total phenolic content was correlated with its reducing power. Hence, It can be inferred that polygonumins A could act as a strong antioxidant agent.

It is well documented that the use of antioxidants may minimize neuronal degeneration and slow the progress of Alzheimer’s disease^35^. Anticholinesterase activity in compounds has also been reported to be related to radical-scavenging activity^36^. Moreover, based on our previous findings on crude extracts of polygonumins A, the compound showed promising anticholinesterase activity^16^. Therefore, we evaluated the anticholinesterase activity of polygonumins A relative to that of tacrine. However, the compound showed only weak anticholinesterase activity at concentrations of up to 2 mg/ml. (Table 2), and the compound did not show potential to serve as an anticholinesterase drug. The IC_50_ for this compound could not be determined due to its low activity; but we managed to determine the IC_20_ value for this compound (1980 µg/ml).

### Molecular docking of polygonumins A, vanicoside A and pepstatin in HIV-1 protease (pdb ID: 3OXC)

**Figure 3:**
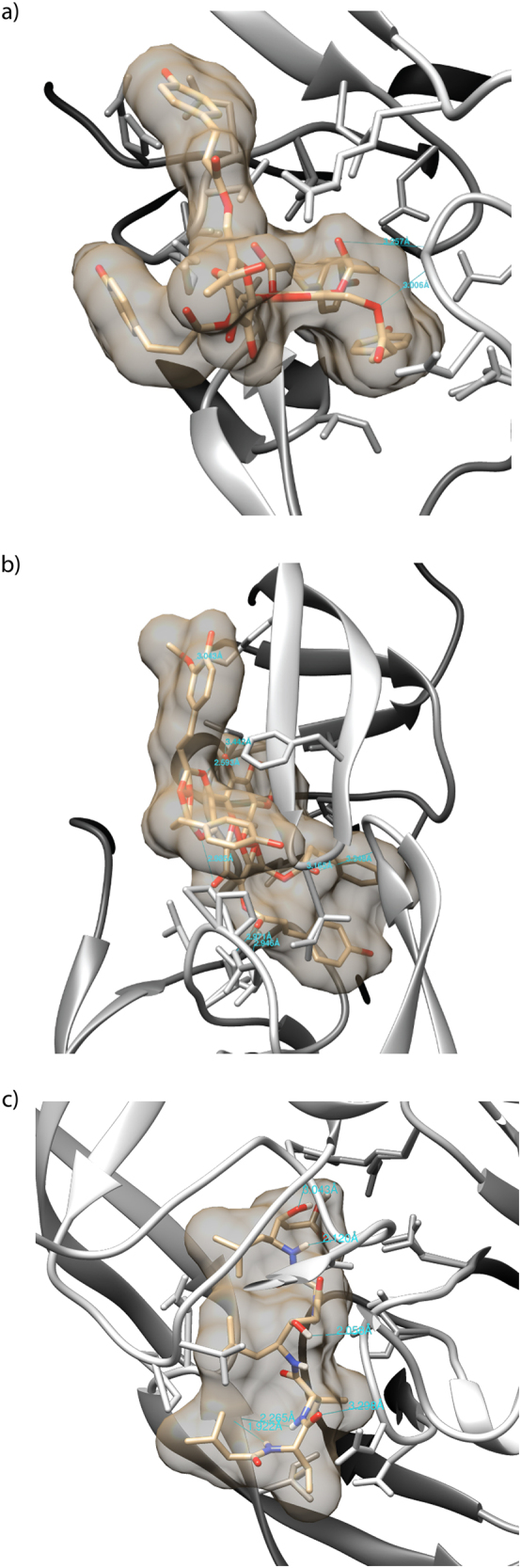
Hydrogen bond interaction of polygonumins A (**a**), vanicoside A (**b**) and pepstatin (**c**) with HIV-1 protease at respective lower binding energy.

**Figure 4:**
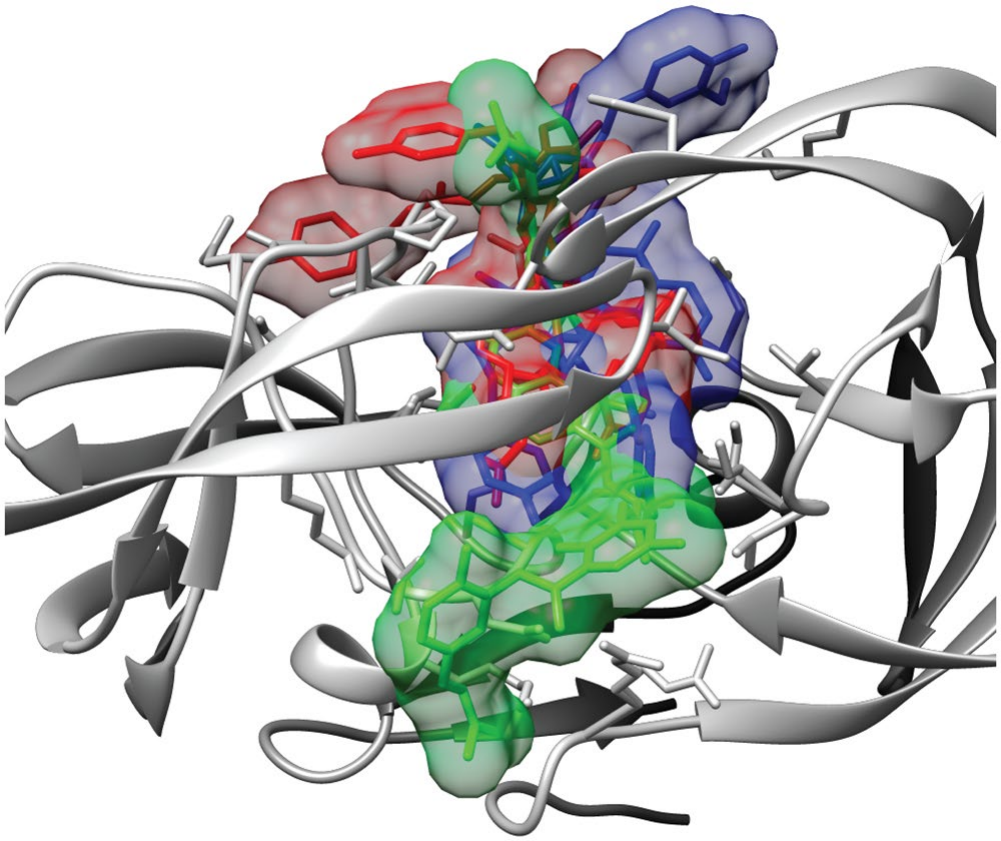
Superimposition of the structures revealed polygonumins A (red), vanicoside A (blue), and pepstatin (green) were docked in a similar binding domain.

Polygonumins A appeared to possess high binding affinity towards HIV protease (pdb ID: 3OXC), as indicated by low binding free energy range of −10.5 to −11.3 kcal/mol (Table 4) recorded in 3 independent runs. The low binding free energies are comparable to those of vanicoside A (−10.5 to −11.7 kcal/mol) docked to the same protein, as illustrated in Fig. 3. Moreover, pepstatin (positive control) exhibited low binding free energies of −8.9 to −9.5 kcal/mol in 3 independent runs. Pepstatin has been used as positive control drug that exhibits anti-HIV properties through enzymatic assay^37,38^, and thus, pepstatin was included as a reference in a comparative analysis of molecular docking to determine the compound’s activity as an anti-HIV protease based on its potential binding affinity. The structures possessing the lowest binding free energy were visualized, and hydrogen bond analysis was conducted using the surface/binding analysis tool in UCSF Chimera version 1.11. The residues involved in hydrogen bond interactions are summarized in Table 5. Moreover, superimposition of the structures showed that polygonumins A, vanicoside A and pepstatin were docked in a similar binding domain (Fig. 4).

**Table 4:** Affinity of polygonumins A, vanicoside A and pepstatin to HIV-1 protease.

	Affinity (kcal/mol)		
	run #1	run #2	run #3
Polygonumins A	−11.3	−10.5	−10.5
Vanicoside A	−10.5	−11.1	−11.7
Pepstatin	−9.5	−9.4	−8.9

**Table 5:** Residues of HIV-1 protease involved in hydrogen bond interactions.

	Residue	Position
Polygonumins A	ASP GLY	29 27
Vanicoside A	ASP LYS ILE ILE THR	30 45 50 150 180
Pepstatin	ASP ASP GLY GLY GLY	29 129 48 127 27

**Figure 5:**
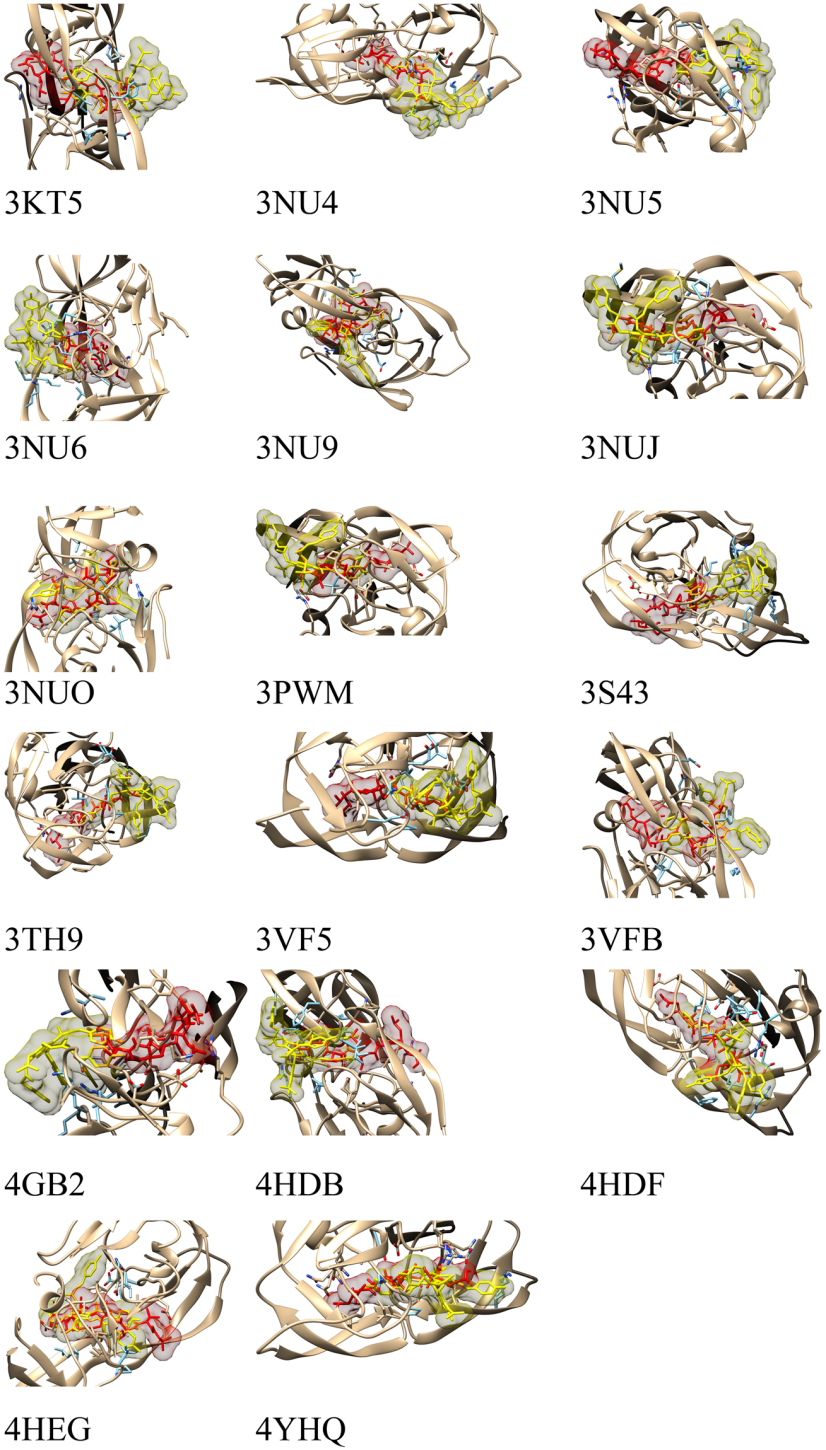
polygonumins A (yellow chain), and pepstatin (red chain) docked in HIV-1 protease mutants (labelled with PDB ID).

Recent studies have reported the anti-HIV activity of several bioactive compounds derived from plant extracts^37,38^ and mushroom^39^. The current study examined the anti-HIV activity of polygonumins A, and the compound’s potential activity was further evaluated to identify the potential interactions of the compound with mutant HIV-1 proteases through molecular docking simulation. Mutant HIV-1 proteases available in the protein databank were downloaded and submitted to docking analysis using VinaAutodock. Polygonumins A was found to engage in potential interactions with mutant HIV-1 proteases with the following PDB IDs: 3PWM, 4HDB, 4HDF, 4HEG, and 4YHQ; the affinities recorded were lower than −10 kcal/mol. The potential interaction was found to be particularly significant for the mutant 4YHQ, for which the affinity was as low as −13.8 kcal/mol. These results are comparable to those obtained for the positive control drug pepstatin, which showed a significant binding affinity towards the mutant HIV-1 proteases. The results are summarized in Table 6. The docked positions of pepstatin and polygonumins A are depicted in Fig. 5. This analysis indicated that polygonumins A possesses the potential to inhibit a range of HIV-1 protease mutants that are currently known to resist available antiretroviral drugs. The potential anti-HIV activities against different protease mutants were predicted based on a complementary fit of polygonumins A with the target HIV protease mutants relative to the positive control drug pepstatin. This approach is well established for identifying potent HIV protease inhibitors^40^, which can be used for drug development in the future.

**Table 6:** Affinities of polygonumins A and pepstatin docked in HIV-1 protease mutants. Residues of HIV-1 protease mutants involved in hydrogen bond interactions with polygonumins A and pepstatin.

Protease Mutant (PDB ID)	Affinity (kcal/mol)		Residues involved in hydrogen bond interaction with ligand : hydrogen bond distance	
	Polygonumins A	Pepstatin	Polygonumins A	Pepstatin
3KT5	−7.3	−8.5	ASP 1029: 3.038 LYS 1045: 2.937 ASP 1030: 3.459	ASP 29: 3.103 GLY 1027: 3.315 GLY 48: 3.244
3NU4	−8.3	−8.6	ASP 29: 3.346 LYS 45: 2.912 LYS 45: 3.176	ASP 29: 2.761 GLY 27: 3.139 GLY 27: 2.985 GLY 127: 3.243
3NU5	−9.1	−8.3	LYS 145: 3.386 LYS 145: 2.800 GLY 148: 3.285 GLU 21: 3.345	VAL 50: 3.225 VAL 150: 3.171 GLY 48: 2.972 ASP 29: 2.859
3NU6	−8.3	−8.3	LYS 45: 3.126 ARG 87: 3.111 ARG 87: 3.039 ARG 8: 2.982 ARG 8: 3.257 ARG 8: 3.335 ARG 8: 3.211	ARG 8: 3.099 ARG 8: 3.006 ASP 29: 3.269 GLY 27: 2.957 GLY 48: 3.089 GLY 27: 2.779
3NU9	−9.3	−8.3	ILE 50: 3.012 GLU 121: 3.158 ASP 130: 3.269 PRO 181: 3.491	ASP 29: 3.087 ILE 150: 3.024 GLY 48: 3.195 GLY 48: 3.166 GLY 48: 2.920
3NUJ	−8.6	−8.1	ASP 30: 3.004 LYS 45: 2.965 ARG 8: 3.222 ILE 50: 3.280 MET 46: 3.620	ASP 29: 3.077 GLY 48: 3.091 ILE 50: 3.078 ASP 30: 2.861 GLY 48: 3.074 GLY 48: 3.084 ASP 25: 3.244
3NUO	−9.3	−8.6	ARG 8: 3.381 ARG 8: 2.944	ASP 29: 2.965 ILE 50: 3.063 GLY 27: 3.018 GLY 48: 3.276 GLY 48: 2.780 GLY 48: 2.892
3PWM	−10.2	−9.8	ASP 30: 2.895 ILE 150: 3.098 ASP 30: 3.371 PRO 181: 3.317	ASP 29: 2.930 GLY 48: 2.880 GLY 48: 3.237 ASP 29: 2.936
3S43	−8.3	−8.4	ARG 8: 2.807 ASP 130: 2.982 LYS 145: 3.279 LYS 145: 3.094 ARG 187: 2.994 MET 146: 3.573	ILE 50: 3.285 GLY 48: 3.113 GLY 48: 3.271 ASP 29: 2.817 GLY 48: 3.283 GLY 48: 2.794
3TH9	−7.5	−8.4	ARG 8: 2.937 GLU 21: 3.160 GLY 48: 3.249	ASP 29: 2.936 ASP 30: 3.356 ASP 29: 3.327 GLY 48: 3.200
3VF5	−9.5	−9.7	ARG 8: 2.962 ARG 8: 3.185 GLY 48: 3.153	ASP 29: 2.991 ASP 29: 3.370 GLY 27: 3.066 GLY 48: 2.894 ASP 25: 3.097
3VFB	−9.4	−9.7	ASP 29: 2.917 ASP 30: 3.371 LYS 45: 2.939 ASN 83: 3.022 ASP 30: 3.335 PRO 81: 3.522	ARG 8: 2.792 ASP 30: 3.031 GLY 27: 3.015 GLY 27: 3.157 ASP 25: 2.967 GLY 48: 2.602 ASP 25: 2.962 ASP 25: 3.130
4GB2	−7.5	−8.3	LYS 45: 3.352 LYS 45: 3.389 ARG 87: 3.383 ARG 87: 2.878 THR 91: 3.220 ASP 29: 3.216	LYS 45: 3.121 GLY 48: 3.035 ARG 8: 3.365 ARG 8: 2.941 GLY 27: 3.034 GLY 48: 2.945 GLY 27: 3.289
4HDB	−11.4	−11.2	ASP 129: 3.175 ASN 130: 2.964 ASN 130: 3.386 LYS 145: 3.088 ASN 130: 3.073	LYS 45: 3.400 GLY 48: 3.100 GLY 48: 3.110 GLY 48: 2.802 GLY 148: 3.261
4HDF	−10.8	−11.2	GLY 48: 3.356 GLY 48: 3.298 ARG 8: 3.335 ARG 8: 3.296 ASP 25: 3.295 GLU 21: 3.436	GLY 27: 3.223 GLY 48: 3.146 GLY 27: 3.341 ASP 25: 3.124 GLY 48: 2.759 ASP 25: 3.462 GLY 48: 2.996 GLY 48: 2.989 ASP 30: 3.165
4HEG	−11.4	−11.3	GLY 48: 3.381 GLY 48: 3.243 ASP 30: 3.125 GLY 49: 3.551	ASP 29: 2.882 GLY 48: 3.135 GLY 48: 3.037 GLY 48: 3.141 GLY 48: 3.166 GLY 48: 3.175
4YHQ	−13.8	−12.2	ARG 8: 3.193 ARG 8: 2.931 VAL 82: 3.170 ASP 29: 3.268 ASN 30: 3.325 ASN 30: 3.184 LYS 45: 3.258 LYS 45: 2.836 GLY 48: 3.261 GLY 48: 2.964 ASN 30: 3.208 ASN 30: 3.478 ASN 30: 3.384	ASP 29: 2.803 GLY 48: 3.098 GLY 48: 2.935 GLY 48: 2.955 GLY 48: 3.178 GLY 48: 2.946 GLY 27: 2.629 GLY 48: 3.292 ASN 30: 2.953

## Experimental procedures

### Plant material

The stem of *P*. *minus* was originally collected from Ulu Yam, Malaysia, and a voucher specimen was deposited in the UKMB Herbarium, Universiti Kebangsaan Malaysia. Specimens were identified by a taxonomist and further confirmed by ITS sequencing^41^. Samples were washed and stored at −80 °C prior to use.

### Isolation of polygonumins A

**Figure 6:**
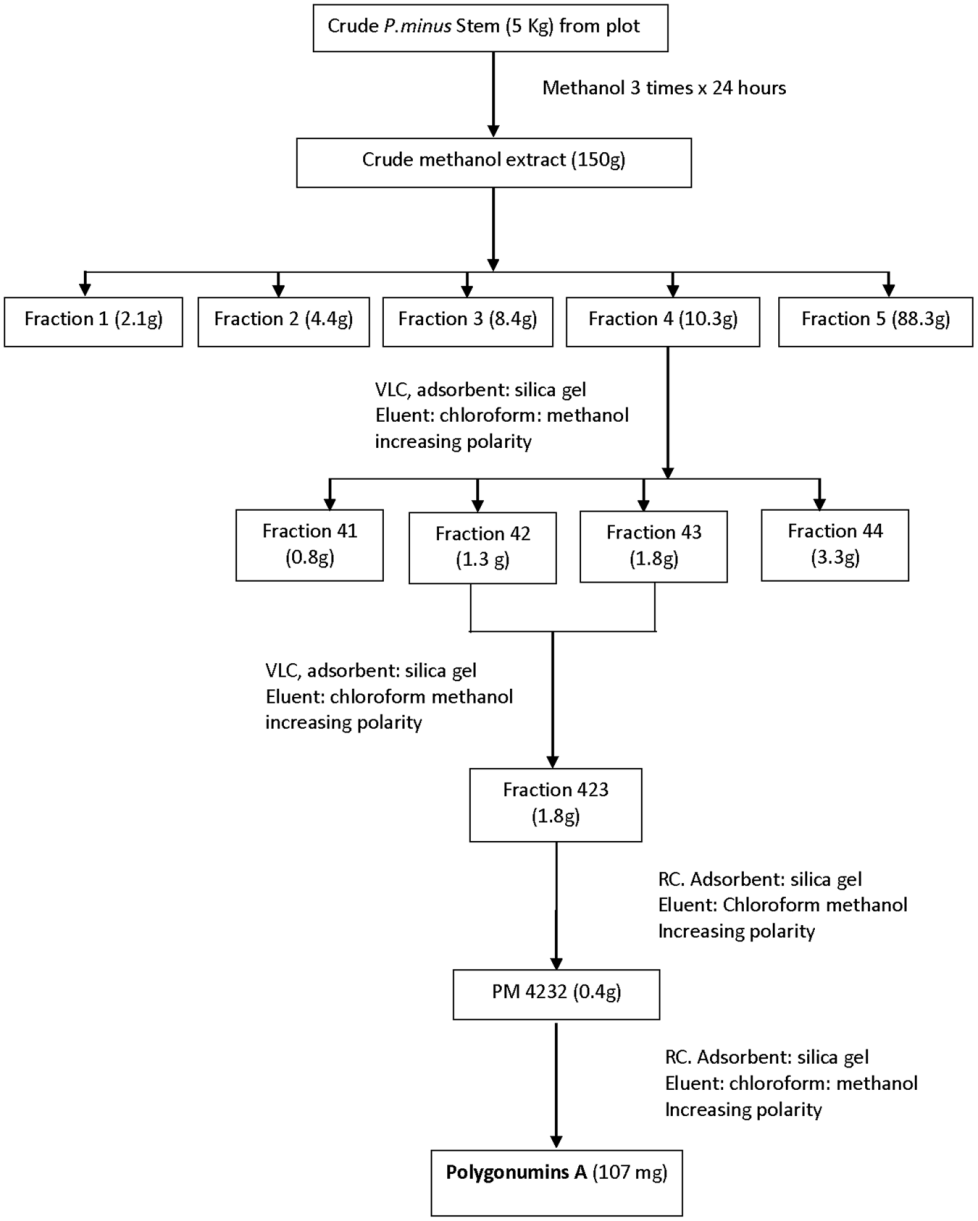
Schematic representation of polygonumins A isolation.

A total of 5 kg of *P*. *minus* stem bark powder (230–250 mesh) was extracted using methanol 3 times for 24 hours each time at room temperature. The extracts were concentrated by a vacuum rotary evaporator at low pressure to yield a dark-green gum (260 g). A total of 150 g of methanol extract was fractionated by vacuum liquid chromatography (VLC) using a column (Φ 10 cm) with silica gel 60PF_254_ (0.063–0.200 mm) as an adsorbent and a chloroform:methanol mixture with increasing polarity (100% chloroform, 90–10%, 80–20%, and MeOH 100%) to yield 5 fractions i.e., F_1_ (2.1 g), F_2_ (4.4 g), F_3_ (8.4 g), F_4_ (10.3 g), and F_5_ (88.3 g), respectively. Based on the spot size produced by thin layer chromatography (TLC), F_4_ was refractionated by the VLC method using a column (Φ 5 cm) with silica gel 60PF_254_ (0.063–0.200 mm) as an adsorbent and a chloroform:methanol mixture (100% chloroform, 90–10%, 80–20%, and MeOH 100%) as an eluent to produce 4 fractions: F_41_ (0.8 g), F_42_ (1.3 g), F_43_ (1.8 g) and F_44_ (3.3 g), respectively. The selected fractions, F_43_ and F_42_ (1.8 g), were combined and further purified by radial chromatography (RC) with silica gel 60PF_254_ containing gypsum as an adsorbent with a chloroform:methanol mixture as an eluent with increasing polarity (95:5% 100% MeOH) to yield Fraction 4232 (0.4 g). Radial chromatography was then performed iteratively using the same eluent mixture to obtain polygonumins A (107 mg) as a white amorphous compound with a melting point of 150.2–152.0 °C. The schematic representation of polygonumins A isolation is summarized in Fig. 6.

### Compound identification

The structure of the purified compound was determined based on spectral data recorded on a Frontier Perkin-Elmer FTIR/NIR (Perkin-Elmer Inc., Norwalk, CT, USA) spectrophotometer and a Bruker NMR 600 MHz Cryo-Probe instrument that could perform 1-D and 2-D NMR measurements (Bruker, Germany). ESIMSs were recorded on a Bruker Daltonics micrOTOF-Q 86 (direct infuse + ve.m). Isolation was carried out by radial chromatography using round glass plates on a Merck Kieselgel 60 PF_254_ (art. no. 7749), and the profile was analysed using aluminium sheets measuring 20 × 20 cm on a Merck TLC silica gel 60 F_254_ with a thickness of 0.25 mm (art. no. 5554) with UV light detection (254 nm) or CeSO_4_ spraying followed by heating.

### Anticholinesterase activity

Inhibition of acetylcholinesterase (AChE) was assessed using the spectrophotometric method developed by Ellman^42^ with slight modifications. Electric eel AchE (electric eel acetyl-cholinesterase, type-VI-S, EC 3.1.1.7, Sigma–Aldrich, St. Louis, USA) and acetylthiocholine iodide (Sigma–Aldrich, Steinheim, Germany) were used as the enzyme and substrate, respectively. Briefly, 125 μl of DTNB (Sigma–Aldrich, Steinheim, Germany) (50 mM Tris-HCl, pH 8, 0.1 M NaCl, 0.02 M MgCl_2_.6H_2_O), 25 μl of AChE (0.2 U/ml), 25 μl of test compound solution in DMSO, and 50 μl of buffer (50 mM Tris-HCl, pH 8, 0.1% BSA) were mixed and incubated at 25 °C for 30 min. DMSO or buffer (25 μl) was added instead of the test compound solution in control experiments. The reaction was then initiated by the addition of 25 μl of acetylthiocholine iodide (0.25 mmol/l), which brought the final volume to 250 μl. The formation of 5-thio-2-nitrobenzoate anion from the enzymatic hydrolysis of acetylthiocholine iodide was monitored based on the absorbance at 412 nm on a 96-well microplate reader (Model 680, Biorad Inc., and Hercules, CA, USA). The reaction rates were calculated from data collected at specific time points over the first 180 s in 20 s increments. Percent inhibition of AChE was determined by the ratio of the reaction rate with the test sample to that with the blank control (DMSO in Tris-HCl buffer, pH 8.0) using the formula (*E* − *S*)/*E* × 100, where *E* is the activity of enzyme with DMSO and *S* is the activity of enzyme with the test sample. The experiments were carried out in triplicate. Tacrine was used as a reference compound.

### Antioxidant activity

#### Free radical-scavenging assa**y**

Radical-scavenging activities were determined by the DPPH (2,2-diphenyl-1-picrylhydrazyl) assay. Various concentrations of the experimental samples were obtained, and the volume was adjusted to 200 μl with ethanol. Approximately 100 μl of 0.5 mM DPPH in methanol was added to the samples or standard (ascorbic acid and gallic acid) in a 96-well plate. After incubation for 30 min in the dark, changes in absorbance at 600 nm were measured on a 96-well plate reader. IC_50_ was calculated as the sample concentration required for a 50% decrease in the absorbance of a control solution of DPPH_._

#### Reducing power assay

Ferric reducing antioxidant power was determined by the direct reduction of Fe^3+^ (CN^−^)_6_ to Fe^2+^ (CN^−^)_6_ and by measuring the absorbance resulting from the formation of the Perls’ Prussian Blue complex following the addition of excess ferric ions (Fe^3+^). The reducing power method was performed as reported by Oyaizu^43^ with slight modifications. Different concentrations of samples (50 µg/ml to 500 µg/ml) were mixed with 2 ml of 0.2 M, pH 6.6 sodium phosphate buffer and 1.25 ml of potassium ferricyanide. The mixture was incubated at 50 °C for 20 minutes. After 20 minutes of incubation, 1.25 ml of trichlorocetic acid (10%) was added. Finally, 0.5 ml of FeCl_3 (0.1%)_ was added to the mixture and incubated for 10 minutes. The intensity of the blue-green colour was measured at 700 nm. Increases in the absorbance of the reaction mixture indicated an increase in the reduction capability. The EC_50_ value (µg/mL) associated with the reducing power-the extract concentration at which the absorbance was 0.5 was calculated from a graph of absorbance at 700 nm against the extract concentration. Gallic acid was used as a positive control.

#### Total phenolic content

The total phenolic content was determined using the Folin-Ciocalteu method^44^. The reaction mixture was prepared by mixing 0.2 ml of sample (1 mg/mL) and 1.5 ml of 10% Folin-Ciocalteu reagent dissolved in water. The mixture was allowed to equilibrate for 5 minutes and then mixed with 1.5 ml 7.5% Na_2_CO_3_ solution. After incubation for 60 minutes at room temperature in the dark, the absorbance of the mixture was read at 725 nm against a blank using a spectrophotometer. The blank was prepared by using DMSO instead of a sample. The sample procedure was repeated for gallic acid at different concentrations, i.e., 0.00, 0.25, 0.50, 0.75 and 1 mg; the results were used to produce a calibration curve. Total phenolic content was calculated as the milligrams of gallic acid equivalent (GAE) per gram of sample (mg GAE/g) by using the gallic acid calibration curve, y = 0.0016× + 0.0295, R² = 0.9548.

### *In vitro* cytotoxicity

#### Cell lines and cell culture

Cells of the lines K-562 (human leukaemia cell line), C33A (cervical cancer cell line), HCT116 (colorectal cancer cell line), and A549 (human lung adenocarcinoma epithelial cell line) were purchased from the American Type Culture Collection. H1299 (human non-small cell lung cancer cell line) and MCF7 (human breast adenocarcinoma cell line) were provided as a courtesy by Prof. Masa-Ikeda of Tokyo Medical and Dental University. Cells were cultured in DMEM media supplemented with 10% foetal bovine serum (FBS) and 1% penicillin and streptomycin. All cell lines were cultured at 37 °C with 5% CO_2_.

#### V79 fibroblast cultures

V79–4 cells were grown in tissue culture flasks using DMEM as the growth medium at 37 °C in a humidified atmosphere of 5% carbon dioxide and 95% air. Cultures were examined daily to ensure they remained healthy. The confluent monolayer was removed by trypsinization, and the number of viable cells was calculated. Cells were seeded into a 96-well plate at a seeding density of 10 000 cells/well and incubated at 37 °C. The test substance was prepared by dilution with an appropriate volume of complete growth medium (DMEM) supplemented with 10% foetal bovine serum (FBS) to obtain the highest working concentration of 100 µg/ml (weight/volume). Procedures were performed aseptically.

The test substance was tested in triplicate at concentrations of 3.125, 6.25, 12.5, 25, 50 and 100 µg/ml. Growth medium from each well of a 96-well plate containing healthy culture was replaced with 200 µl of the test substance and controls respectively. The cultures were then incubated for 24 hours at 37 °C in a humidified atmosphere of 5% carbon dioxide and 95% air.

#### *In vitro* cytotoxicity assay

Cells were plated in 96-well microplates and cultured for 24 h. The test compounds were dissolved in DMSO and added at different concentrations (100, 50, 25, 12.5, 6.25, 3.13 µg/ml) to the plate in triplicate. The cytotoxicities against K-562 and H1299 cell lines were measured using a colorimetric MTT (3-(4,5-dimethylthiazol-2-yl)−2,5-diphenyltetrazolium bromide) assay. Exponentially growing cells were harvested and suspended in DMEM, and a 100 µl cell suspension was added to a 96-well plate. After 24 h incubation at 37 °C with 5% CO_2_, the cells were treated with varying concentrations of the test compounds (100 µl). The medium was removed, and cells in each well were incubated with PBS containing 1 mg/ml MTT for 24 h at 37 °C with 5% CO_2_. DMSO (100 µl) was added to each well to dissolve the insoluble formazan crystal, and plates were incubated for 4 h at 37 °C for complete solubilization of formazan. The level of coloured formazan derivative was analysed on a microplate reader using the results at wavelengths of 570 nm and 630 nm as references^45^. The percent viability of cells was calculated using the following equation:

$$ \% \mathrm{viability}=\,\frac{{\rm{Absorbance}}\,{\rm{of}}\,{\rm{treated}}\,{\rm{cells}}}{{\rm{Absorbance}}\,{\rm{of}}\,{\rm{cells}}\,{\rm{in}}\,{\rm{solvent}}}\times 100$$

Percent viability was plotted versus compound concentration, and IC_50_ values at which the compound showed 50% inhibition of tumour cell proliferation were calculated using Microsoft Excel. The compound was tested at each concentration in triplicate.

#### HIV-1 protease fluorogenic assay

The HIV-1 Protease Inhibitor Screening Kit (Fluorometric) from Biovision Incorporated (Milpitas, CA, USA) was used to measure the inhibitory effect of each sample on HIV-1 protease activity. Pepstatin A (1 mM) was used as a known standard for HIV-1 protease inhibition, and 1% DMSO was used as a solvent control. The assay was performed according to the manufacturer’s instructions. Briefly, each sample (1 mg/ml) was incubated with HIV-1 protease enzyme at room temperature for 15 minutes. Then, the HIV-1 protease fluorescent substrate was added. The fluorescence (excitation/emission = 330/450 nm) in kinetic mode over a period of 90 minutes at 37 °C was determined using a PerkinElmer EnSpire plate reader.

### Statistical analysis

One-way ANOVA at 95% confidence level was used for statistical analysis followed by Dunnett’s test in relation to control and standard.

### Molecular docking

The structure of wild-type HIV-1 protease (PDB ID: 3OXC) was downloaded from the protein databank for molecular docking. The protein model was prepared by removing all non-standard residues that included antiviral drug saquinavir, sulfate ion and formic acid in their structure. Water molecules were removed from the structure and hydrogen atoms added using AutoDockTools-1.5.6 prior to conversion of the file format to PDBQT for docking analysis. A grid box with dimensions of 50 × 40 × 40 A^3^ was generated with HIV-1 protease as the centroid using AutoDockTools-1.5.6. Vanicoside, pepstatin, and polygonuminss were docked in the HIV-1 protease using AutoDockVina 1.1.2^46^. Docking of the molecules was repeated in three independent runs. Structures were visualized and hydrogen bond interactions were further analysed using UCSF Chimera version 1.11^47^.

## Conclusion

Polygonumins A, a new compound isolated from *P*. *minus*, showed promising anticancer and HIV protease inhibition activities. This compound has been filed for a Malaysian patent (PI 2014700594). In summary, our investigation provided promising results supporting the potential use of *P*. *minus* in the treatment of cancers such as leukaemia and colorectal and breast cancer and as an anti-HIV agent. Hopefully, these leads can be taken up for the further development of anticancer and anti-HIV drugs.
